# Multidrug efflux in Gram-negative bacteria: structural modifications in active compounds leading to efflux pump avoidance

**DOI:** 10.1038/s44259-024-00023-w

**Published:** 2024-03-16

**Authors:** Dominik Gurvic, Ulrich Zachariae

**Affiliations:** 1 Computational Biology, School of Life Sciences, University of Dundee, Dundee, DD1 5EH UK; 2 Biochemistry and Drug Discovery, School of Life Sciences, University of Dundee, Dundee, DD1 5EH UK

**Keywords:** Drug discovery, Data processing

## Abstract

Gram-negative bacteria cause the majority of critically drug-resistant infections, necessitating the rapid development of new drugs with Gram-negative activity. However, drug design is hampered by the low permeability of the Gram-negative cell envelope and the function of drug efflux pumps, which extrude foreign molecules from the cell. A better understanding of the molecular determinants of compound recognition by efflux pumps is, therefore, essential. Here, we quantitatively analysed the activity of 73,737 compounds, recorded in the publicly accessible database CO-ADD, across three strains of *E. coli* – the wild-type, the efflux-deficient *tolC* variant, and the hyper-permeable *lpxC* variant, to elucidate the molecular principles of evading efflux pumps. We computationally investigated molecular features within this dataset that promote, or reduce, the propensity of being recognised by the TolC-dependent efflux systems in *E. coli*. Our results show that, alongside a range of physicochemical features, the presence or absence of specific chemical groups in the compounds substantially increases the probability of avoiding efflux. A comparison of our findings with inward permeability data further underscores the primary role of efflux in determining drug bioactivity in Gram-negative bacteria.

## Introduction

Gram-negative (GN) bacteria are responsible for the majority of highly or extremely drug-resistant bacterial infections. For the most critically resistant pathogens, drug treatment strategies are now limited to few remaining options, and therefore new or improved antibacterials are urgently needed^1–4^. However, only a small number of antibacterial drugs are currently under development, whose activity is moreover strongly biased towards Gram-positive (GP) bacteria^5,6^. The architecture of the GN cell envelope, consisting of two lipid membranes which enclose the periplasmic space, represents the major obstacle preventing sufficient drug activity in GN bacteria^7–9^. By contrast, GP bacteria only possess a single membrane. The chemical determinants for enhanced drug permeation across the GN cell envelope remain largely unclear, which substantially hinders the design of new drugs with GN activity^10,11^.

**Fig. 1:**
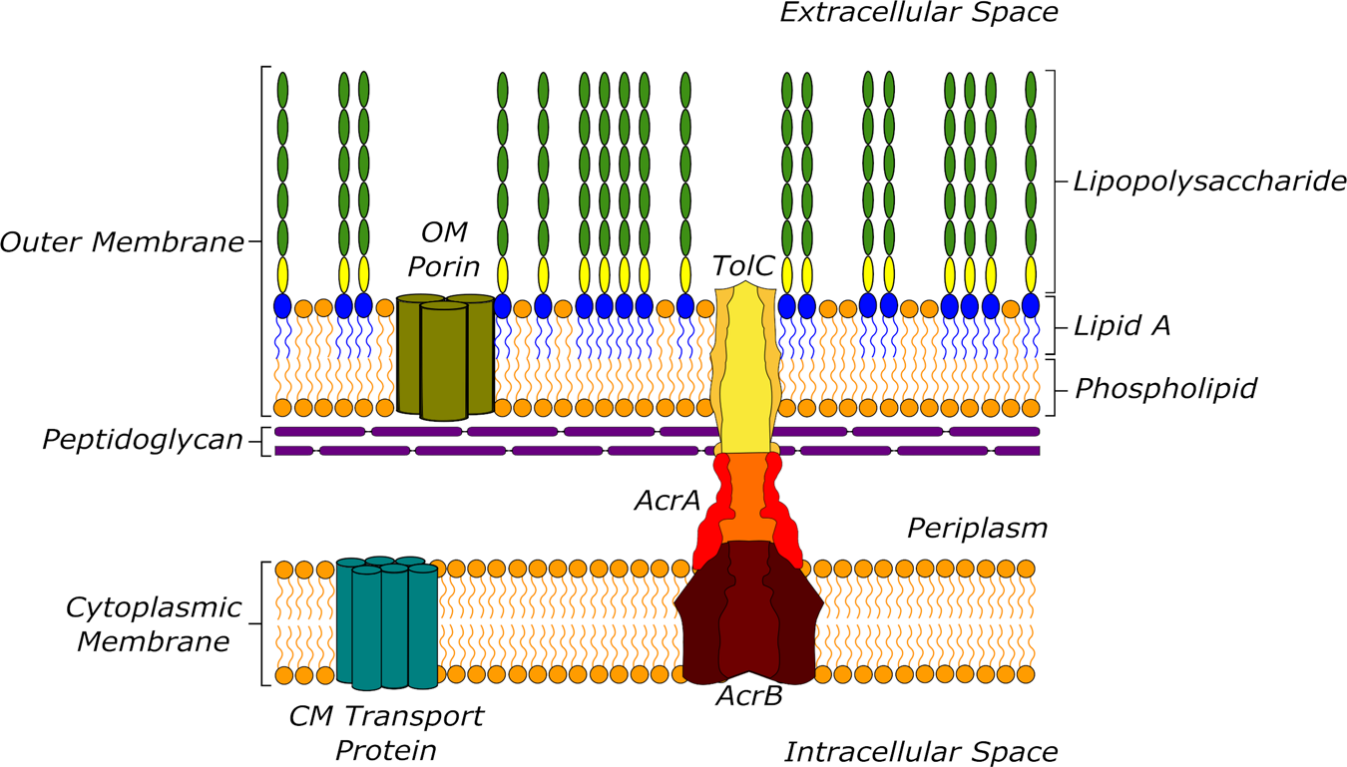
Diagram of the Gram-negative cell envelope. The outer and inner membranes enclose the periplasm with the in-lying peptidoglycan layer. Porins provide entry passageways for polar molecules into the periplasm. Tripartite efflux pumps (here: the *E. coli* AcrAB-TolC complex) span the inner and outer membranes and efficiently expel drugs from the periplasm.

The two lipid membranes in the GN cell envelope, the outer membrane (OM) and cytoplasmic membrane (CM) contain key proteins which play an important role in drug permeability (see Fig. 1). Inward drug permeation across the OM is thought to proceed primarily via porin proteins, whose pores possess specific geometries and are highly polar with a strong transversal electrostatic field, limiting the chemical space available for permeating molecules^12,13^. Porins can undergo mutations, and their expression level can be down-regulated to enhance resistance^10,14–16^. For many GN bacteria, however, active drug efflux is thought to be a major driver of intrinsic and acquired drug resistance^17–19^. In particular, GN bacterial tripartite efflux pumps, which span both the CM and the OM and the periplasm, efficiently recognise and expel most drugs from the bacterial periplasm before they are able to reach their therapeutic targets (Fig. 1)^20–22^.

Tripartite efflux pumps are driven by electrochemical gradients across the CM or by ATP hydrolysis in the cytoplasm^17,23^. They consist of an active pump protein in the CM, an adaptor protein in the periplasm, and an outward conduit protein in the OM, with varying stoichiometries^21^. The spectrum of efflux substrates is notoriously broad, and a range of different pumps operate in parallel^17,24,25^. In Enterobacteriaceae, however, including the best understood GN bacterium *Escherichia coli*, only one gene encodes an OM conduit protein, TolC^26–28^. Consequently, all parallel efflux pathways based on tripartite efflux pumps can be inhibited by the deletion of this gene in *E. coli*^21^.

So far, the understanding of the molecular basis for OM permeation and active efflux remains insufficient, although several studies have highlighted the collective physicochemical properties of molecules with GN activity. GN-active molecules were found to be generally slightly larger and more hydrophilic (lower *logD*) than inactive molecules^29^, while small compounds with particularly low *logD* from the AstraZeneca collection were shown to be least susceptible to be effluxed^30^. Given that many compounds are active in the cytoplasm, the further requirement to cross the CM adds another level of complexity to simplified physicochemical design rules concentrating on OM permeability, however, and molecular details need to be taken into account^31^. Richter et al. have for instance recently revealed the importance of positively charged primary amine moieties for entry into GN bacteria^32^. Focusing on drug efflux, El Zahed et al. used activity data measured on a non-public compound library of 314,000 molecules in wild-type (WT) and efflux-compromised *tolC E. coli* to construct a machine learning model to predict the susceptibility to efflux from combinations of collective molecular descriptors. Amongst the key descriptors determining efflux susceptibility discovered in that study were resonant structure count and hydrophilicity (*logD*)^33^.

In the present work, we conducted a large-scale, data-driven chemical analysis of the experimental compound activity in different *E. coli* strains obtained from the publicly accessible database CO-ADD (Community for Open Antimicrobial Drug Discovery)^34,35^. We focused in particular on the individual chemical structure of 73,737 compounds acting on WT *E. coli* (strain ATCC 25922), the efflux-deficient variant *tolC* (strain MB5747), and the OM-permeable variant (*lpxC*, strain MB4902). In *lpxC* *E. coli*, the lipopolysaccharide (LPS) content of the otherwise poorly permeable outer leaflet of the OM is reduced, generating porin-independent permeation pathways^36–38^.

Comparing the activity of each compound across the three variants allowed us to gain insight into the balance of the two major factors underpinning low drug uptake in GN organisms, active efflux and OM permeation, and potential differences in their chemical and physical determinants. Using Matched Molecular Pair Analysis (MMPA), we identified small molecular changes that are repeatedly observed to convert a given compound from an efflux pump substrate into an efflux pump evader while at the same time not being prone to inward permeability issues. These molecular modifications and physicochemical guidelines may help medicinal chemists and drug designers to rationally enhance drug uptake in GN bacteria, which has so far represented a major obstacle to the development of GN-active antibiotics.

## Results

### Compound classification

We sourced experimental growth inhibition (GI) data, reflecting the activity of each investigated molecule in WT as well as the *tolC* and *lpxC* variants of *E. coli*, from the public-domain CO-ADD project database (www.co-add.org)^34^. Since there can be substantial variation in assay conditions and results between different laboratories, we opted for consistent, single-source data recorded in-house at CO-ADD, which we deemed optimal for subsequent analysis. The data sets consist of GI values obtained as optical density values (*OD*_600_) after treatment of bacterial cultures with the compounds. The values are expressed as a percentage of inhibition by normalising the test compound *OD*_600_ values to those obtained for bacteria without inhibitors (0% GI) and media only (100% GI). Note that values outside the 0–100% range can occur as, for example, some compounds could enhance growth. The compound dataset is diverse and does not focus on particular structural scaffolds, when compared to a diverse high-throughput screening library (Supplementary Fig. 1)

**Fig. 2:**
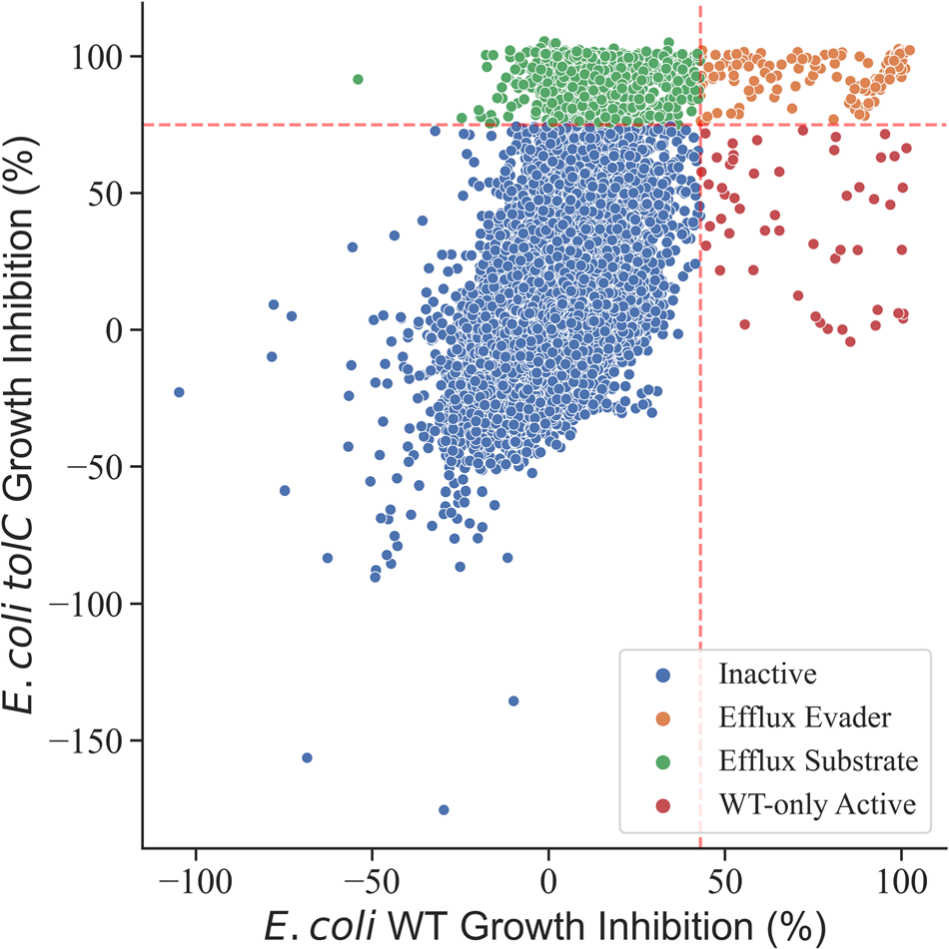
Distribution of compound activity in WT and *tolC E. coli*. Graph displaying the experimentally determined growth inhibition values for each investigated compound in WT (*x*-axis) vs. *tolC E. coli* (*y*-axis). The thresholds for classification are shown as red dashed lines, classification results are shown in colour code. (Additional distribution of WT and *tolC* activity: Supplementary Fig. 1).

**Fig. 3:**
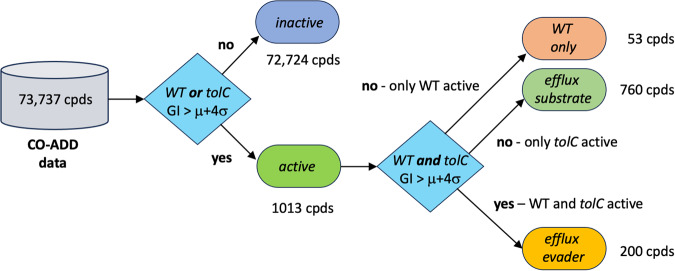
Flow chart of the compound classification process. The chart shows the protocol we used to define efflux substrates and efflux evaders in addition to WT-only actives and inactives according to growth inhibition (GI) data from WT and *tolC E. coli*.

To classify any given compound as active, we applied a strict criterion to prevent noise by setting the threshold to a level that exceeds the mean compound activity in the tested set (*μ*) by four standard deviations (*σ*) (*GI* ≥ [*μ* + 4*σ*]). We then classified compounds as efflux substrates or efflux evaders as follows: Compounds active against WT *E. coli* at the 4*σ* level that at the same time showed activity against *tolC* *E. coli* at the same level were classed as efflux evaders. The rationale for this classification was that, as judged from the GI data, compound activity did not depend on the function of TolC-dependent efflux mechanisms. Efflux substrates were then identified in the compound data by being active in the *tolC* strain but inactive in WT *E. coli*, such that the activity of these compounds was likely to be suppressed by TolC-dependent efflux. Accordingly, compounds inactive in both WT and *tolC* *E. coli* were classed as generally inactive, while compounds that were active in WT but inactive in *tolC* were classed as WT-only active. The activity thresholds and distribution of the compounds are shown in Fig. 2 and the classification scheme we used in Fig. 3. The classification of initially 73,737 compounds resulted in 200 efflux evaders, 760 efflux substrates, 53 WT-only actives and 72724 inactives.

As mentioned above, it is highly challenging to identify compounds with sufficient activity against GN bacteria^39^. The data set is, therefore, strongly biased towards the inactive class. However, the rigorous curation performed here yielded a sufficiently large data set of particularly high quality, containing the most relevant compound classes for the present study, efflux substrates and evaders. The significance of the smallest class, WT-only active, is intuitively less clear. It is conceivable, however, that WT-only active compounds interact directly with parts of the efflux machinery of the WT or the *tolC* gene.

An interesting question, also addressed in our recent work^40^, is the differential activity of compounds in GN *vs*. GP bacteria. We therefore further analysed the activity of the compound dataset against *Staphylococcus aureus* as a typical GP pathogen.

Based on the same activity threshold, we found that 1275 molecules are active against WT *S. aureus* within the set of 73,737 compounds, given availability of *S. aureus* data. As expected, the number of active molecules is thus much greater in that set than the number of actives against WT *E. coli*, where we identified only 275 active compounds in total. This confirms further the notion that OM permeability and efflux are major obstacles for GN bioactivity.

Additionally, a breakdown of differential activities in *S. aureus* and *E. coli* shows that the largest number of compounds in the data set with any antibacterial activity are active only against *S. aureus* (1099 compounds). Only 176 compounds are active against both *E. coli* and *S. aureus*. This number is greatly increased, however, in the *lpxC*, and especially the *tolC* mutants (Supplementary Table 1, Supplementary Fig. 3). Of note, there are 99 compounds that only show activity in *E. coli* but not *S. aureus*, which could indicate that these molecules target elements of the OM such as the LPS. However, this is a small set of compounds by comparison.

The *E. coli* data classified according to efflux characteristics then underwent a further curation step accounting for the inward permeability of the compounds across the OM. Similar to efflux, the compounds were classified into OM permeating, OM non-permeating, WT-only active and inactive molecules by evaluating activity differences between WT *E. coli* and the OM-hyperpermeable *lpxC* variant (see Supplementary Figs. 4, 5, and 6). Out of the 760 compounds classified as efflux substrates, 206 were additionally found to be OM non-permeable. Similarly, out of the 200 efflux evaders, 186 were also classified as OM permeators, while the other 14 were found to be WT-only active with respect to OM permeation.

As we ultimately aimed to identify chemical strategies to evade active efflux pumps, in this first step, we removed known non-permeating efflux substrates from further analysis. This was done in order to prevent inferring rules from compound pairs that may improve efflux evasion, but still exhibit low inward permeability in either case, despite the chemical changes. Such compounds, we deemed, would not form a strong basis for further antibiotic development. Although, again, it is challenging to rationalise activity only in the WT, the 14 WT-only active compounds were also not investigated further to avoid any convolution of efflux with other effects. After accounting for OM bias, we therefore took 186 efflux evaders and 554 efflux substrates into the further steps of analysis (for a complete list of efflux evaders and substrates, see Supplementary Dataset 1).

Notably, examining the relative impact of low OM permeability *vs.* active efflux on the bioactivity of compounds, we found that a compound’s inactivity in WT can be explained by insufficient inward permeation across the OM in 369 cases, while it can be linked to active efflux in 760 cases. Hence, according to the data set investigated here, active efflux contributes to low GN bioactivity at a ratio of 2:1, as compared to overcoming the OM barrier. This shows that, indeed, active efflux is the predominant factor in determining low drug uptake across the GN cell envelope.

### Collective physicochemical and structural differences between efflux substrates and evaders

We next examined if physicochemical or structural rules can be established to differentiate efflux evaders from substrates. Many efflux systems in Gram-negative bacteria recognise a notoriously promiscuous spectrum of substrates^20,41^. Due to the existence of parallel efflux pathways and several substrate binding sites in some efflux pumps, they bind and expel drugs of a multitude of different chemotypes^17,42^. We thus first investigated if there are potential gaps in the recognition of molecules based on their physicochemical parameters.

In earlier work, it has been suggested that compounds with whole-cell activity in GN bacteria are more hydrophilic, often zwitterionic, smaller, and have a larger polar surface area than their GN-inactive counterparts^18,29–31,33,43,44^. Recent studies have also concluded that compound rigidity, represented by a number of rotatable bonds below five in the molecule, aids accumulation in GN bacteria^32,39^. Additionally, structural elements such as various amines, thiophenes, and halides have been linked with increased GN bacterial permeation^32,40^.

In many previous studies, the shift towards increased hydrophilicity and rigidity, as well as lower MW, has been explained by the geometric and physicochemical constraints imposed by OM permeation via porins^12,32^. For efflux in particular, recent investigations have also suggested that hydrophobic compounds are more likely to be actively expelled, whereas small hydrophilic or charged compounds and polar zwitterions have a higher probability of avoiding efflux^30^. However, the authors note that “simply designing polar compounds was not sufficient for antibacterial activity and pointed to a lack of understanding of complex and specific bacterial penetration mechanisms"^30^. Recently, additional features contributing to the efflux susceptibility of compounds in *E. coli* have been identified, including planarity and a greater degree of elongation with limited branching^33^.

**Fig. 4:**
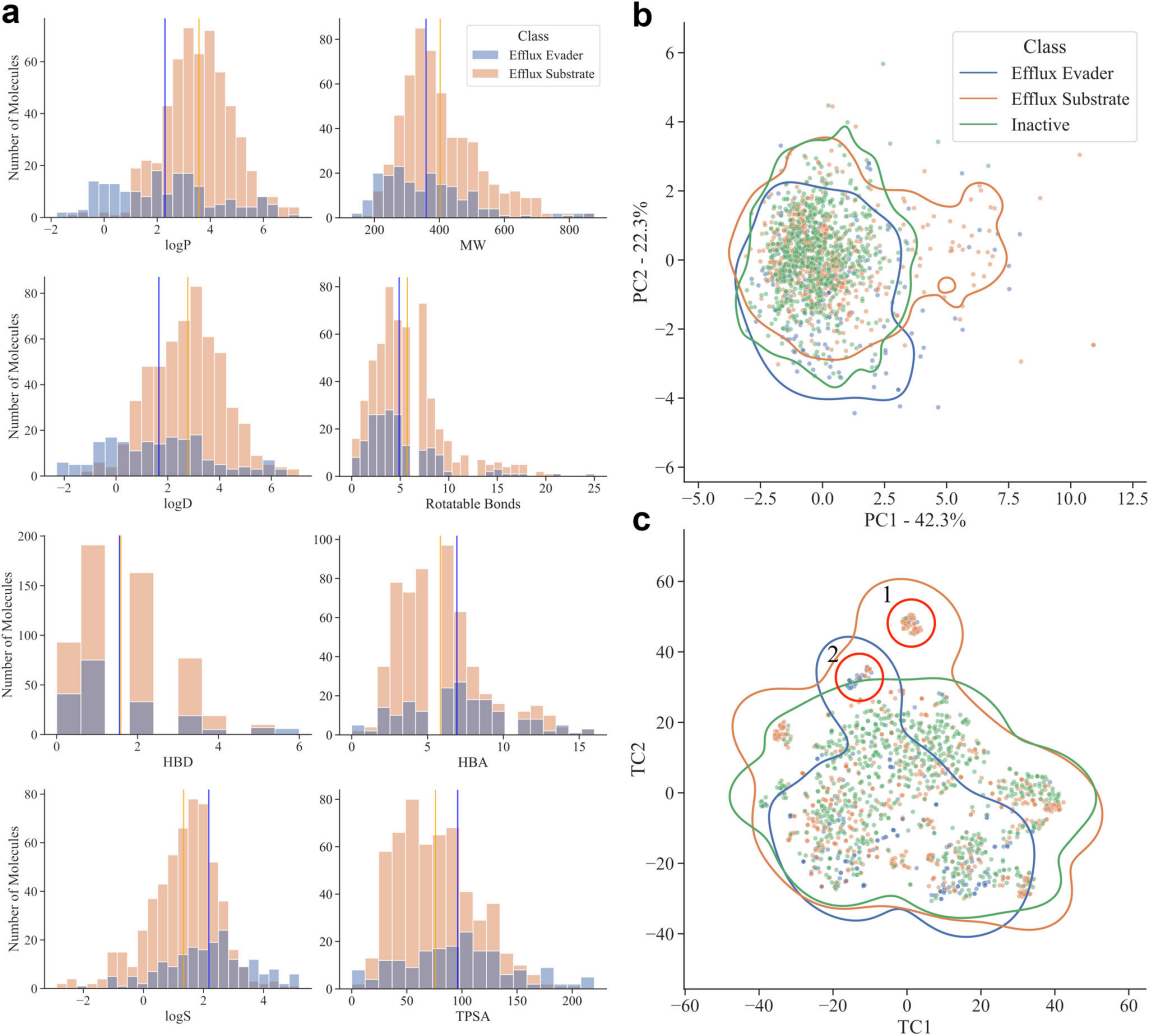
Physicochemical analysis of efflux evaders and substrates. **a** Distribution of eight key physicochemical features for efflux evaders (blue) *vs.* efflux substrates (orange). The mean values of each distribution are shown as vertical lines. **b** Principal component analysis using the same set of physicochemical features for efflux evaders, substrates and inactive compounds. The first two principal components cover 64.6% of the variance. **c** Comparison of the chemical similarity space occupied by efflux evaders, substrates and a sample of inactive molecules by t-SNE analysis. Two distinct clusters are marked by red circles. The contour lines in (**b**) and (**c**) were determined using a kernel density estimator and drawn at a density value of 0.05.

Based on the large dataset investigated in the present study, we calculated physicochemical descriptors of the molecules for efflux substrates, efflux evaders, and a sample set of 500 inactive molecules for comparison. They include molecular weight (MW), hydrophobicity (*logP* and *logD*_7.4_), topological polar surface area (TPSA), solubility (*logS*), the number of hydrogen bond acceptors and donors, and the number of rotatable bonds (Fig. 4a). We found that the efflux evaders in our investigated data set, indeed, tend to be more hydrophilic (*logP* and *logD*), possess a larger polar surface area, and have greater solubility in water. They also have, on average, a slightly lower MW, whereas only a small difference is observed in their flexibility as compared to efflux pump substrates. However, the distributions of these features for the two classes show significant overlaps in most cases, making them poorly separable (Fig. 4a, Supplementary Fig. 8). The best separation is seen for *logP* and *logD*, where nearly no efflux substrates are observed with a *logP* below 1 or a *logD* below 0, that is, the probability of evading efflux is substantially increased for very hydrophilic compounds.

A principal component analysis (PCA) of the entire set of calculated physical and chemical molecular features, by contrast, showed that efflux evaders and substrates occupy largely the same space. The largest contributor to principal component 1 (PC1) was the topological polar surface area (TPSA), while for principal component 2 (PC2), it was compound hydrophobicity (*logP*; Fig. 4b). Importantly, a sample of 500 inactive compounds also displayed a substantial overlap with efflux evaders and substrates in the physicochemical feature space. This indicates that compound activity and interactions with efflux pumps may not be sufficiently explainable by a combination of collective physicochemical features alone and that, additionally, molecular structural information must be taken into account.

We, therefore, next used t-distributed Stochastic Neighbour Embedding (t-SNE) to reduce the dimensionality of the compound structural data to a two-dimensional representation according to their structural similarity (Fig. 4c). The t-SNE plot shows that efflux evaders, substrates, and inactives again occupy largely the same similarity space, demonstrating that structurally similar molecules interact differently with the efflux pumps. Of note, however, are two small separated clusters of structurally similar molecules at the top of the t-SNE plot (Fig. 4c, Clusters 1 and 2). Cluster 1 consists of 40 compounds, three of which are efflux evaders, while 37 are efflux substrates. Cluster 2 displays a ratio of 23 efflux evaders *vs*. 9 efflux substrates, none of which possess similarity to any of the 500 sampled inactive molecules (the structures belonging to the two clusters are shown in Supplementary Dataset 2, the maximum common substructures characterising each cluster in Supplementary Fig. 9). A large proportion of the compounds in cluster 2 contain a carboxylic acid moiety. However, we were not able to identify any molecular transformations within this cluster that convert an efflux substrate into an efflux evader by adding a carboxylic acid group (for details on molecular transformations, see below).

In summary, we conclude that it is – similar to the observations we made for the physicochemical parameters – challenging to predict the interaction of the three compound classes with efflux pumps solely on the basis of collective molecular features such as structural similarity. We, therefore, next investigated if more fine-grained differences on the level of small structural substitutions may be decisive for the recognition of compounds by efflux pumps.

### Molecular transformation of compounds between the inactive, substrate and evader classes

**Fig. 5:**
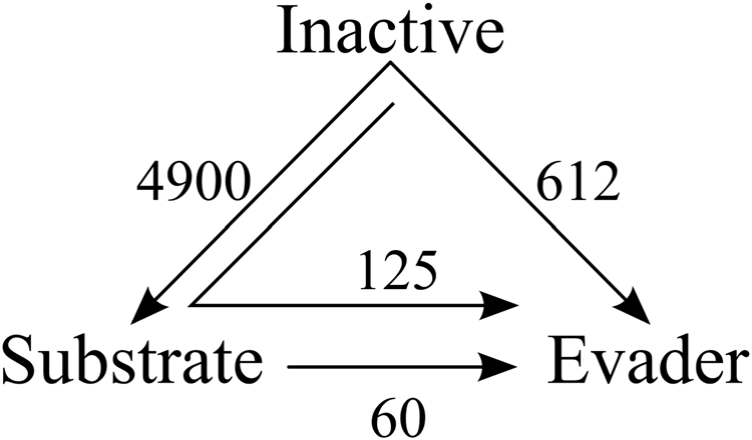
Molecular transformations resulting from matched molecular pair analysis. The MMPA transformations connect compound pairs converting similar compound cores from inactive to efflux substrate, inactive to efflux evader, and from substrate to evader (arrows). The numbers of independent transforms are shown next to each arrow. The arrow inside indicates a double transform, from inactive to substrate and further to an evader, connecting compound triplets.

We used matched molecular pair analysis (MMPA) to analyse small structural changes that convert compounds with a common core between the three compound classes; inactives, efflux evaders, and efflux substrates (Fig. 5). The analysis yielded a set of 4900 substrate transforms, in which 2053 inactive compounds are transformed into 349 substrates, as well as a set of 612 transforms in which 397 inactive compounds are converted into 77 efflux evaders. Note that in all cases, the number of transforms exceeded the total number of the compounds since, in many cases, multiple compounds are transformed into the same substrate or evader, while individual compounds can also be transformed into multiple substrates or evaders (see Table 1). Overall, due to the smaller number of efflux evaders in the initial data set, there are fewer transforms leading to evaders than to substrates, while the number of inactive compounds exceeds both substrates and evaders.

**Table 1:** Matched molecular pairs.

Type of Transform	Inactives	Substrates	Evaders	No of Transforms
Inactive into Substrate	2053	349	N.A.	4900
Inactive into Evader	397	N.A.	77	612
Substrate into Evader	N.A.	26	24	60
Inactive into Substrate into Evader	52	23	15	125

All transformations from MMPA, together with the number of unique compounds from each class within the transforms. Multiple transforms can occur between similar compounds, such that the number of transforms exceeds the number of classified compounds.

Central to the main focus of our study, 60 of the identified transforms converted 26 substrates into 24 evaders. Moreover, 125 double transforms linked 52 unique inactive compounds to 23 substrates and, further, to 15 evaders by consecutive molecular substitutions, connecting compound triplets rather than pairs. Each individual sequence of transforms in these 125 examples contained an identical core and three replacement moieties attached to the same location on the core, resulting in members of the three different compound classes.

**Fig. 6:**
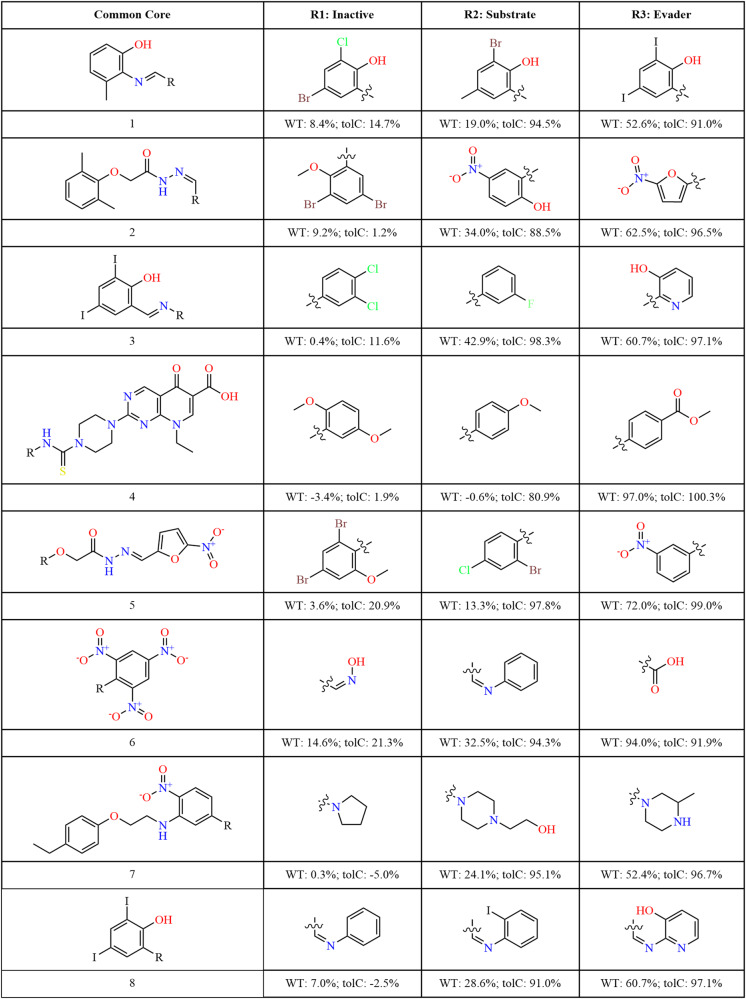
Transforms linking compound classes. Exemplar transformations converting inactive molecules into efflux substrates and further into efflux evaders by small structural modifications between these compound triplets. The first column shows the compound core shared by all three molecules in the triplet, and the following three columns show the substitutions resulting in inactive, substrate and evader compounds. `R' on the common core structure indicates the location of the molecular substitutions in column 1; `R1' is the substitution required for inactives, `R2' for substrates, and `R3' for efflux evaders. Each compound is labelled with its associated growth inhibition values measured for WT *E. coli* and the *tolC* variant, respectively.

Figure 6 shows eight exemplar double transforms (all transforms are provided in the Supplementary Materials, see Supplementary Dataset 3). The associated WT and *tolC* activity values highlight the large impact of small substructural changes on the compounds’ bioactivity and their interaction with efflux pumps.

### Transforming efflux substrates into efflux evaders

Focusing on molecular transforms that turn efflux substrates into evaders, we searched for recurring patterns in which the addition or removal of chemical moieties led to this conversion (Table 2). We found that within the transforms, the addition of pyridine was seen to transform efflux substrates into efflux evaders in 22 individual cases. Likewise, adding primary, secondary, or tertiary amines (either aliphatic or aromatic amines) converted substrates into evaders in 16 independent transformations. In addition to these nitrogen-containing groups, adding *α*-halogenated carbonyl groups was found to turn substrates into evaders in four cases. Conversely, looking at chemical moieties whose removal promoted efflux evasion, in addition to the more exotic iodo-group, we found aromatic alcohols (10 repeats), quaternary ammonium cations (7 repeats), ketone and aldehyde groups (altogether 6 repeats), as well as ether groups (4 repeats). These findings suggest that specific chemical groups can enhance the recognition of a compound as an efflux substrate.

**Table 2:** Efflux-related functional groups.

Moieties	Effect	Repeats
Pyridine	Positive	22
1^∘^ /2^∘^ /3^∘^ aromatic and aliphatic amine	Positive	16
Alpha-halogenated carbonyl	Positive	4
Iodo-moieties	Negative	11
Aromatic alcohols	Negative	10
Quaternary ammonium	Negative	7
Alkyl and aryl ketones and aldehydes	Negative	6
Ether	Negative	4

Moieties whose addition to or removal from a common core promotes a compound’s avoidance of efflux. The moieties have a positive effect on efflux evasion when their addition results in an evader and a negative effect when their removal results in an evader. The number of transforms in which the same moiety and effect are observed is shown as repeats.

The overall picture that emerges from transforming efflux substrates into evaders is that replacement of oxygen-containing functional groups with nitrogen-containing groups aids in efflux evasion, with the exceptions of quaternary ammonium cations (negative correlation with efflux evasion) and *α*-halogenated carbonyls (positive correlation with efflux evasion). This effect may be due to specific molecular features of the substrate binding sites in the bacterial efflux pumps.

Notably, the largest contributor to the transformation of compounds from inactives to both substrates and evaders is the nitro group. The polar nitro moiety appears in 62 evader compounds and in 138 substrate compounds, such that the addition of the nitro moiety to an inactive compound can lead to both evaders and substrates. It is noteworthy, however, that a remarkable enrichment of evaders is observed amongst the nitro-containing active compounds, occurring in 62 out of 186 analysed evaders (33%).

To ascertain if the moiety exchanges identified within the transformations conformed to the general shifts in the physicochemical parameters observed for efflux evaders *vs.* substrates (see Fig. 4), we examined their average change amongst all transformations. Table 3 shows that the evaders were smaller than their substrate counterparts, with an average decrease of around 37 Da (−10.7%) in the transformations. Evaders were also more hydrophilic than substrates, with a *logP* and *logD* decrease of −33.3% and −38.1%, respectively. On average, the number of rotatable bonds decreased, albeit by a small percentage (−5.8%), indicating that evaders tend to be more rigid. The solubility, *logS*, increased (15.5%) in line with a lowered *logP*. A raised TPSA (21.8%) indicates a gain in potential polar interactions, again in agreement with a decreased *logP*. The largest magnitude increase was seen for the number of hydrogen bond donors (28.7%) in addition to a smaller increase in hydrogen bond acceptors (18.0%). This translates into an increase of nearly one additional hydrogen bond acceptor in evaders, on average, as compared to substrates.

**Table 3:** Physicochemical features associated with efflux evasion.

PC feature	Substrates	Evaders	Change	Bulk Change	OM Permeation
MW	348.93	311.53	−10.7%	−10.95%	+0.18%
*logP*	2.58	1.72	−33.3%	−36.6%	−13.24%
*logD*	2.26	1.40	−38.1%	−40.79%	−21.15%
Rot. bonds	3.24	3.05	−5.8%	−15.2%	−8.81%
*logS*	2.45	2.83	+15.5%	+63.9%	+7.62%
TPSA	71.72	87.37	+21.8%	+27.14%	+1.59%
HBA	5.05	5.96	+18.0%	+19.14%	+5.64%
HBD	0.94	1.21	+28.7%	−1.72%	−3.96%

Average shift in physicochemical features in the molecular transforms converting compounds from substrates to evaders (change), as compared to the shift of physicochemical features in all compounds classed as substrates and evaders (bulk change) and the shifts observed for all OM non-permeable *vs.* OM-permeable compounds.

The specific molecular transformations converting efflux substrates into evaders reproduced the bulk shifts in physicochemical parameters observed for all substrates *vs.* evaders (Fig. 4, Table 3), with the exception of the number of hydrogen bond donors, where a marked increase was seen for the transform pairs. Overall, the major changes in these markers point to increased hydrophilicity and polarity as a key to enhancing the probability of evading efflux pumps. Taken together, these findings lead to an important conclusion: While a change in collective parameters, such as an increase in hydrophilicity, can be accomplished in various different ways by substituting hydrophobic moieties with polar moieties, our analysis shows that the *type* of the hydrophilic group is the key difference between obtaining an efflux pump substrate or an efflux pump evader. More specifically, with the exception of quaternary ammonium cations, the addition of nitrogen-containing groups aids efflux evasion, whereas it is the removal of polar oxygen-containing moieties that yields the same effect, despite an overall average gain of hydrophilicity.

By comparing the set of physicochemical changes of efflux substrates *vs*. evaders with the physicochemical changes of OM non-permeating *vs.* OM-permeating compounds (derived from GI values measured in WT and *lpxC E. coli*), we further found that the major collective parameters associated with improved GN bioactivity showed a much closer link to efflux evasion than to inward permeability across the OM. This result further supports the notion that efflux is the main contributor determining whole-cell bioactivity in GN bacteria and that evading efflux pumps is thus key to the design of GN-active antibacterials.

## Discussion

Although drug resistance occurs in both GP and GN bacteria, GN bacteria have a much higher propensity for displaying critical or extreme resistance^1–4^. GN bacterial infections are more difficult to treat, and drug development against GN bacteria is faced with substantial hurdles, the major one being insufficient drug uptake across the GN cell envelope^10,11^.

In the present study, we focused on the role of active drug efflux in low drug uptake. GN efflux systems expel compounds of a wide range of different chemotypes. The determinants of compound recognition by efflux pumps and, especially, their avoidance, are only sparsely understood^17,24,33,45–47^. We, therefore, investigated what can be learned from the differential activity of 73,737 compounds from the CO-ADD database in three different variants of the GN bacterium *E. coli*; the WT, the efflux-deficient *tolC* strain, and the OM hyper-permeable *lpxC* strain. Many early studies of drug permeation and efflux in GN bacteria were based on smaller numbers of compounds, which additionally often showed considerable similarity to each other^30,48^. Only recently, the efflux-propensity of a large compound library of 314,000 molecules has been investigated and used to construct a machine-learning model to predict efflux susceptibility^33^. Furthermore, in recent years, large compound activity databases such as CO-ADD have become publicly accessible, enabling the analysis of big data sets with greater statistical power in the public domain^34^.

It is important to note that our study is, at present, agnostic to the specific targets of action of each compound; especially as most compounds are exploratory and have not been studied intensely in terms of their mechanism of action. This means that the molecules may act inside the cytoplasm, in the CM, the periplasm, or even externally on the LPS. Active efflux may thus play a different role with respect to different targets. However, efflux will affect all compounds except for those acting in the OM or LPS. Moreover, most existing antibiotics have targets either in the cytoplasm or periplasm and so it is likely that active efflux is relevant for the majority of molecules in the dataset we investigated.

Our analysis suggests that the physicochemical features commonly associated with increased general GN bioactivity of a compound increase the probability of avoiding active efflux. These features include enhanced hydrophilicity, a larger polar surface area, high solubility in water, and increased H-bonding potential^29–31,33^. In many previous studies, the increased bioactivity of these compounds had been rationalised by assuming that polar molecules are more readily able to pass through the highly charged interior of porin channels in the OM^12,16,32,39^. In the data set we analysed, active efflux appears to be the major contributor to low GN bioactivity. Our analysis suggests that the physicochemical characteristics underpinning GN activity serve to promote the evasion of efflux pumps. Comparing the relative importance of efflux vs. OM permeability in reduced WT bioactivity, about two-thirds of the compounds in our dataset that are active in either the efflux-deficient *tolC* strain or the OM hyper-permeable *lpxC* strain but not the WT of *E. coli* are effluxed, whereas one-third are poorly permeable across the OM. Moreover, analysis of data from the *lpxC* strain shows that enhanced OM permeability is less correlated with changes in physicochemical features such as polar surface area or hydrophilicity than general GN bioactivity or efflux.

For comparison, we also addressed the efflux-susceptibility of existing GN-active antibiotics and of highly similar molecules. Within the investigated dataset, we identified 36 active compounds with a Tanimoto similarity of between 100% and 85% to a list of marketed GN-active compounds. Indeed, 33 out of these 36 compounds are classified as efflux evaders according to our criteria. Only two compounds are efflux substrates, while one compound is WT-only active. This finding further highlights the importance of avoiding efflux for GN bioactivity.

Since it had been noted previously that the design of polar compounds is not sufficient for antibacterial activity^30^, we furthermore investigated distinct chemical transformations that aid in efflux evasion. A more complex picture of efflux avoidance emerged upon a detailed structural analysis of compound pairs, in which small chemical modulations transform efflux substrate compounds into efflux evaders. Specifically, avoiding recognition by efflux pumps is linked to certain chemical modifications that conform to the physicochemical guidelines noted above, but not to alternative molecular substitutions that may have the same effect on the physicochemical parameters. For example, adding any type of amine groups and/or pyridine, and in this way increasing hydrophilicity, is linked with the majority of molecular transformations that convert efflux substrates into evaders. By contrast, it is necessary to remove quaternary ammonium cations to achieve a similar effect. Likewise, hydrophilic oxygen-containing groups, including ketones, aldehydes, ethers, and aromatic alcohols, promote the recognition of compounds by efflux pumps.

A notable limitation of our study is the absence of GI data for porin-deficient *E. coli* mutants. Primary amines, for example, have been associated with an enhanced ability to traverse general porins^32^. Although our analysis suggests that efflux evasion is the primary factor in the increased bioactivity of the compounds containing pyridine, any type of amine, and nitro groups, we cannot currently rule out that, especially in the case of primary amines, an interplay between increased influx rates and reduced efflux exists. This would lead to similar observations, as ultimately, the balance between influx and efflux determines the bioavailability of the compounds. Further experiments with porin-deleted mutants are needed to fully clarify this question.

Overall, our analysis leads to the following conclusions: With regard to uptake through the GN cell envelope, the data suggests that the bioactivity of a compound in the GN bacterium *E. coli* is mainly driven by its propensity to be an efflux substrate. Strongly hydrophilic compounds with large polar surface areas and high solubility are more likely to evade efflux; however, our analysis indicates that specific molecular modifications in the direction of increased hydrophilicity are required to escape efflux, while others have the opposite effect. In particular, nitrogen-containing functional groups, including amines, pyridine, and the nitro moiety, are connected with a higher probability of evading efflux, even though we cannot exclude that a complex interplay between porin permeation and efflux leads to the increased bioactivity of some of these compounds.

Further limitations of our study are the restriction to compounds tested in *E. coli* and its variants, as well as the inevitable bias between a large number of inactive compounds compared to a relatively small number of actives, of which only a subset is classified as efflux pump evaders. We selected a strict criterion for the compound activity to obtain a curated data set with minimal noise.

Additionally, there are limitations with regard to the *E. coli* mutants used for our data analysis. The hyperpermeable *lpxC* mutant, for instance, primarily facilitates the diffusion of hydrophobic compounds across the OM, while polar or charged molecules still require porin-dependent pathways. This may have an impact on our compound classification protocol. Also, not all efflux in *E. coli* is TolC-dependent. Future studies are necessary in the future to address the chemical determinants of drug efflux within other GN pathogens and a broader range of variants from the WT.

## Methods

### Data source

The CO-ADD microbial growth inhibition database contains information on ~100k compounds, from both academic and industry sources, screened against five bacterial pathogens, their mutants, as well as two fungal pathogens (*Escherichia coli* WT - ATCC 25922, *Escherichia coli* *lpxC* - MB4902, *Escherichia coli* *tolC* - MB5747, *Klebsiella pneumoniae* - ATCC 700603, *Acinetobacter baumannii* - ATCC 19606, *Pseudomonas aeruginosa* - ATCC 27853, *Pseudomonas aeruginosa* PΔ7 - PAO397, methicillin-resistant *Staphylococcus aureus* - ATCC 43300, and the fungal pathogens *Cryptococcus neoformans* - ATCC 208821 and *Candida albicans* - ATCC 90028). Inhibition of bacterial growth was determined by measuring light absorbance at 600 nm (*OD*_600_) after treatment with compounds. The percentage of growth inhibition (activity) was calculated for each assay, using media only as a negative control and bacteria without inhibitors as a positive control, on the same plate as references. The growth inhibition database was downloaded from the CO-ADD website www.co-add.org^34^. For further information on the screening approach, see ref. ^35^

### Determining the threshold for activity

To analyse this dataset, we assigned an activity threshold that separates inactive compounds from active ones. Based on a combination of activity/inactivity of each compound in each of the *E. coli* strains, i.e., WT, *tolC* and *lpxC*, the following classes were assigned with respect to efflux pump interaction and outer membrane permeability. We defined the threshold for an active compound as an activity value at least four standard deviations (*σ*) greater than the mean (*μ*) activity value for all compounds (≥[*μ* + 4*σ*]). More specifically, the mean value of the percentage of growth inhibition after treatment with a concentration of each compound in WT E. coli is *μ* = 4.1%, and the standard deviation is *σ* = 9.7%. Hence, the compounds were classed as WT active if their activity is ≥43.0%.

The determination of the activity threshold in the *tolC* strain was performed as follows: The mean activity value was *μ* = 6.6% with standard deviation *σ* = 17.1%, giving a threshold for active compounds in this efflux deficient strain of ≥75%. Lastly, for the *lpxC* strain, the activity threshold to classify compounds into OM-permeable and non-permeable was obtained via an activity mean of *μ* = 5.6% and a standard deviation of *σ* = 14.0%. Hence, the compounds were classed as *lpxC* active if ≥61.8%. All the means, standard deviations and activity thresholds in their respective strains are summarised in Table 4.

**Table 4:** Mean and standard deviation of compound activity levels.

*E. coli* strains	*μ* [%]	*σ* [%]	Activity threshold [%]
WT	4.1	9.7	43.0
*tolC*	6.6	17.1	75.0
*lpxC*	5.6	14.0	61.8

Determining the activity threshold for three strains, in order to classify the compounds into actives or inactives, where the activity threshold is ≥[*μ* + 4*σ*].

Note that the threshold of at least four standard deviations is higher than those traditionally applied for this type of analysis of between two and three. However, this strict criterion allowed us to reduce potential noise from false positives.

### Physicochemical properties and principal component analysis

A principal component analysis (PCA) was carried out to reduce the dimensionality of the space spanned by all physicochemical descriptors and visualise potential differences between efflux evaders, efflux substrates, and inactive compounds in a reduced-complexity space^49^. RDkit was used for the calculation of PC features^50^. Features were standardised to avoid issues due to scaling and used in linear dimensionality reduction by applying the PCA method in scikit-learn version 1.2.0^51^. The first two principal components described a sufficient 64.6% of the variance. Physicochemical features contributing the most to the first two principal components were: the hydrophobicity measure (*logP* and *logD*), solubility (*logS*), topological polar surface area, number of rotatable bonds, molecular weight, hydrogen bond acceptors, and hydrogen bond donors.

### t-SNE and molecular similarity

To perform the t-SNE analysis, we applied the implementation of t-Distributed Stochastic Neighbour Embedding in scikit-learn^52^. Initially, RDkit was employed to compute Morgan fingerprints for each molecule, using a radius of 2 and generating 2048-bit fingerprint vectors^50,53^. Subsequently, we performed t-SNE analysis with the Jaccard distance metric to reduce the data points from 2048 dimensions to two dimensions for visualisation^54^. The Jaccard distance is also referred to as the Tanimoto distance, and it is defined as *Tanimoto distance = 1 - Tanimoto similarity*. Therefore, the proximity between points in the t-SNE plots reflects the Tanimoto similarity of the corresponding molecules, with greater distances indicating lower Tanimoto similarity. We explored the perplexity parameter, which defines the number of nearest neighbours considered in the calculations and settled on a value of 50; the remaining parameters were used at their default values^51,55^.

### Matched molecular pair analysis

To identify small molecular differences between compounds with and without efflux pump interactions, we carried out a matched molecular pair analysis (MMPA). Matched molecular pairs (MMPs) were generated by adapting the method from Dalke et al.^56^. The results from MMPA yield pairs of similar compounds (compound_A and compound_B) and a small structural change between those compounds (transform), while most of the molecule (core) remains the same (Table 5)^57^. Compound_A contains the so-called left-hand-side (LHS) of the transformation, the moiety attached to the core that is replaced, while compound_B contains the ‘new’ right-hand-side (RHS) chemical moiety.

**Table 5:** Anatomy of a transform.

compound_A	Transform	compound_B
core + LHS	LHS → RHS	core + RHS

A single MMPA transform consists of: Compound_A, built from a core and the left-hand-side (LHS); the transform then refers to changing the LHS to a right-hand-side (RHS) resulting in Compound_B, which maintains the same core as Compound_A, but with a new RHS attached.

We curated all matched molecular pairs such that the transform (each LHS and RHS) contained fewer atoms than the common core and only up to two cut locations (Table 5). This step was taken to to restrict the size, number, and location of the transforms to chemically meaningful replacements that are practically feasible. Transforms with at least three repeats were retained for further analysis.

### Reporting summary

Further information on research design is available in the Nature Research Reporting Summary linked to this article.

## Supplementary information

SUPP Material

Supplementary Dataset 1

Supplementary Dataset 2

Supplementary Dataset 3

Reporting Summary

## Data Availability

All the underlying code, together with all key datasets and working examples of generating results, can be accessed via Github: https://github.com/domgurvic/efflux_evaders_and_substrates.
