# Supplementary material for: Multidrug efflux in Gram-negative bacteria: structural modifications in active compounds leading to efflux pump avoidance
Source: NPJ Antimicrob Resist. 2024 Mar 16;2:6. doi: 10.1038/s44259-024-00023-w (PMC11721645; doi:10.1038/s44259-024-00023-w)
Supplement: Supplementary file 1 — SUPP Material [file 44259_2024_23_MOESM1_ESM.pdf]

Supplementary Material for: Multidrug Efflux in Gram-Negative Bacteria: Structural Modifications in Active Compounds Leading to Efflux Pump Avoidance

Dominik Gurvic* and Ulrich Zachariae*

E-mail: dgurvic@dundee.ac.uk; u.zachariae@dundee.ac.uk

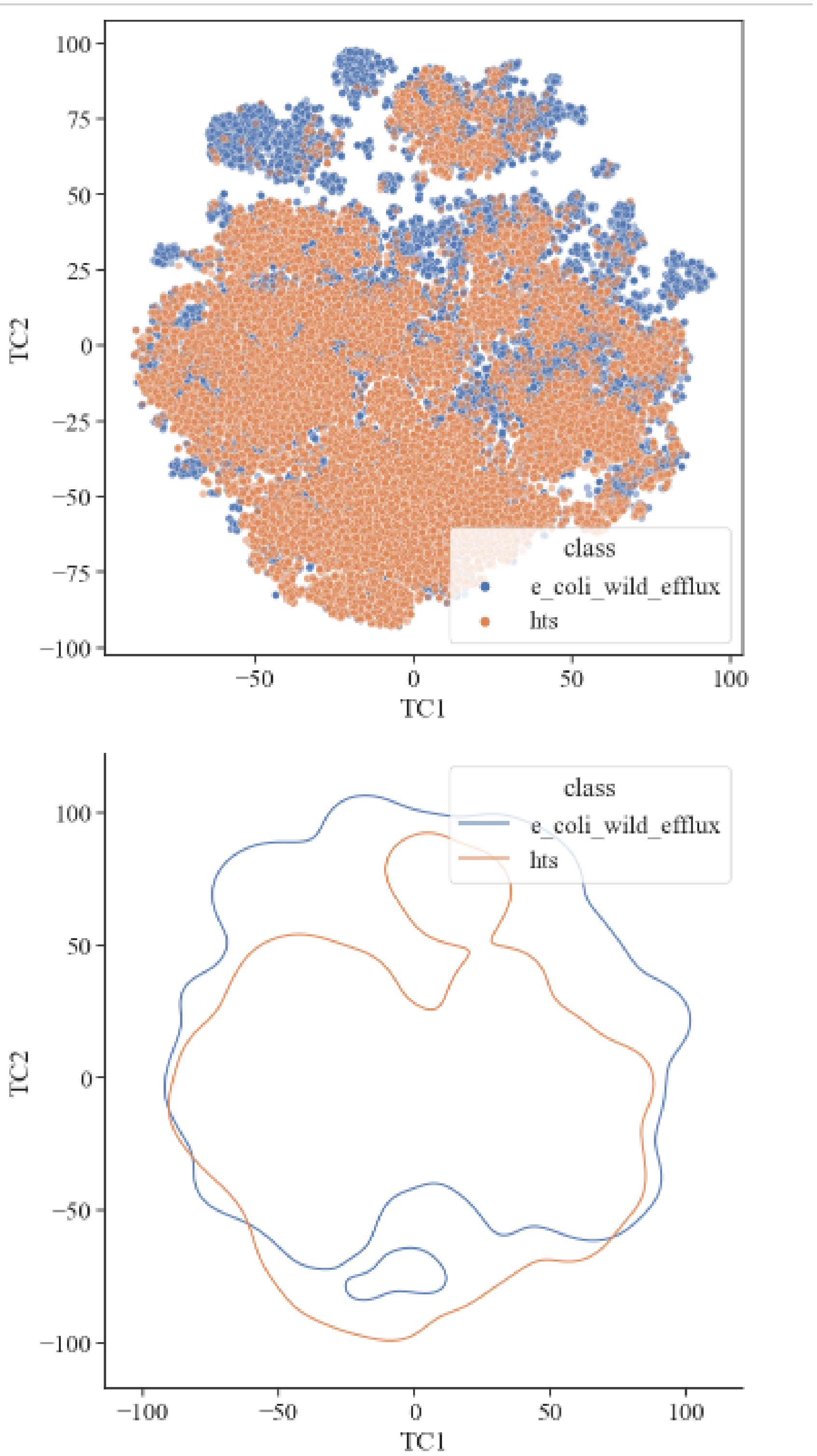

Supplementary Figure 1. Comparison of the diversity of the compounds in the analysed dataset with the high-throughput screening compound library from Enamine. An equal number of compounds was selected randomly for comparison.

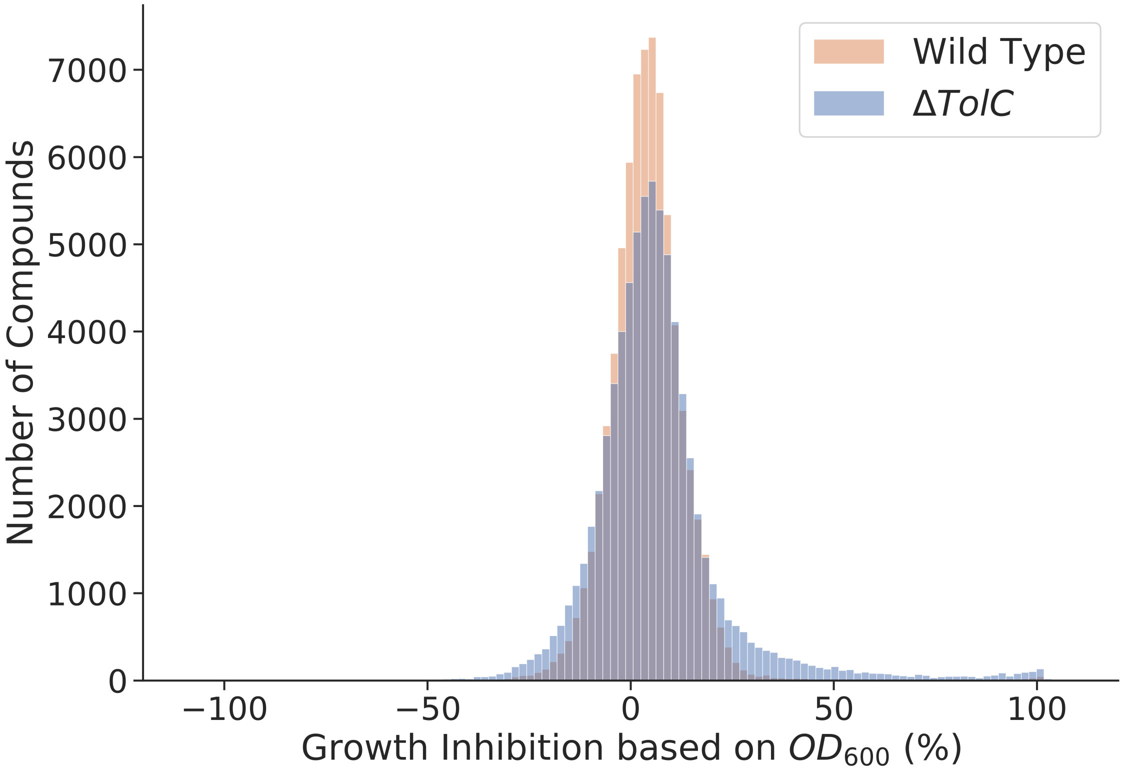

Supplementary Figure 2. Distribution of the growth inhibition activity of $\sim 74k$ compounds from the CO-ADD database tested against WT (orange) and efflux-deficient *tolC E. coli* (blue). The distribution for *tolC* data exhibits a lower peak and a distinct tail stretching to the right. This suggests that a number of compounds show greater activity in the efflux-deficient strain. A paired t-test between two distributions returned a p-value below 0.05, confirming that removing efflux pumps significantly affected growth inhibition.

Supplementary Table 1. Differential actvity of compounds against GP *S. aureus* and *E.coli.*

Activity type	WT	lpxC	tolC
E. coli inactive / *S. aureus* active	1099	927	695
E. coli active / *S. aureus* active	176	319	498
E. coli active / *S. aureus* inactive	99	270	460

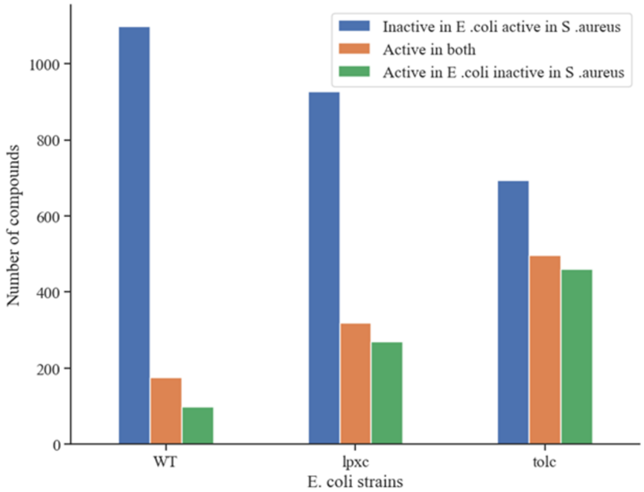

Supplementary Figure 3. Bar graph showing the effect of the *E. coli* mutations in achieving similar activity levels as in the GP pathogen *S. aureus*

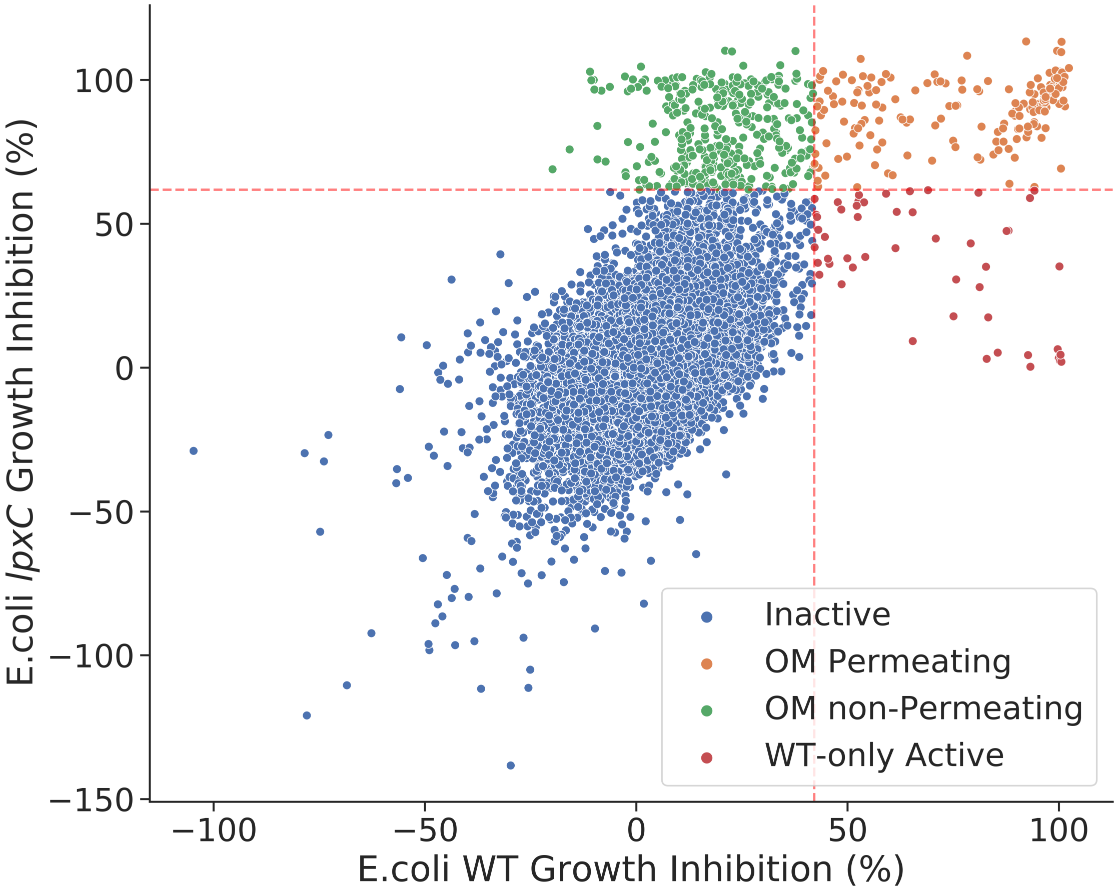

Supplementary Figure 4. We used the same activity thresholds as for WT vs. *tolC E. coli* to analyse GI activity in the WT and hyper-permeable *lpxC E. coli* strains. Inactives (blue) are identified as compounds that show activity below the threshold in both WT and *lpxC* strains. OM permeating compounds (orange) are active above the threshold in both WT and *lpxC*. OM non-Permeating compounds (green) are inactive in WT but active in *lpxC*. WT-only active compounds (red) are active only in the WT. The broken red lines show the activity thresholds at $GI > \mu + 4\sigma$ for both strains.

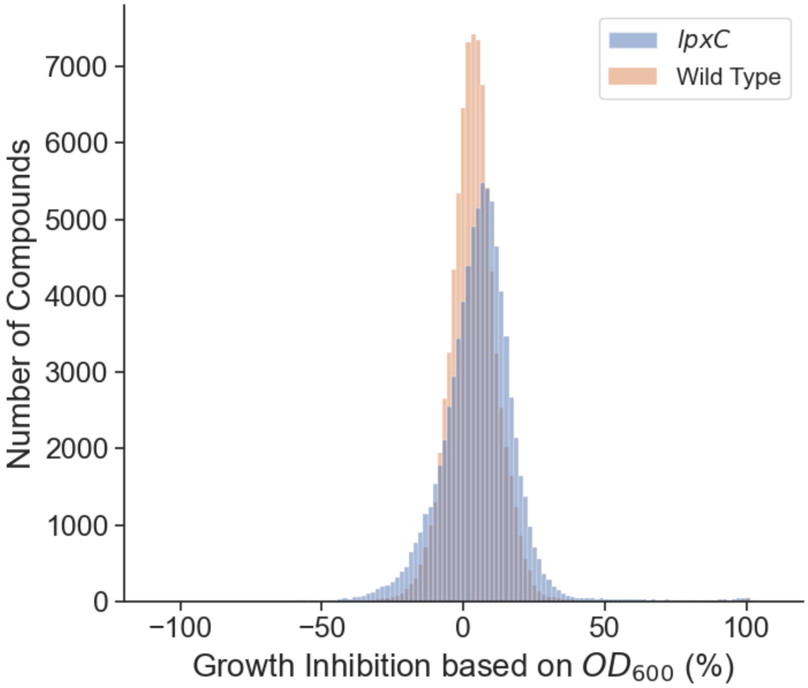

Supplementary Figure 5. Distribution of the growth inhibition activity of $\sim 74k$ compounds from the CO-ADD database tested against WT (orange) and hyper-permeable *lpxC E. coli* (blue). The mean of the distribution for *lpxC* data is clearly right-shifted, suggesting that, on average, the compounds show greater activity in the hyper-permeable strain. A paired t-test between two distributions returned a p-value below 0.05, confirming that OM permeability significantly affected growth inhibition.

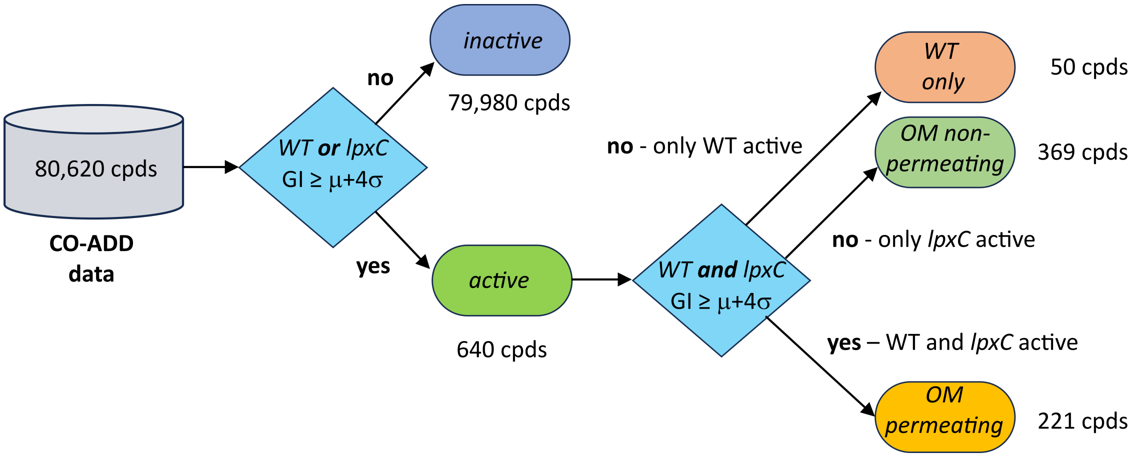

Supplementary Figure 6. Flow diagram of the classification scheme for OM permeable and non-permeable compounds, in addition to inactive and WT-only active compounds.

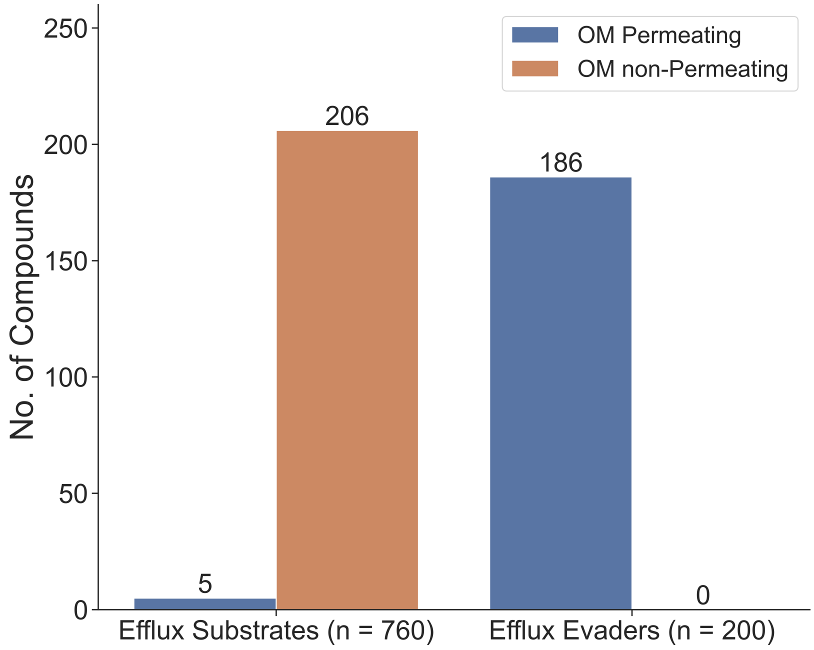

Supplementary Figure 7. Additional effect of OM permeation on efflux evaders and substrates. Out of 760 efflux substrates, 206 compounds are also classed as OM non-permeable, which means their WT inactivity could be caused by a combination of these features. Out of 200 efflux evaders, 186 are also classed as OM permeable.

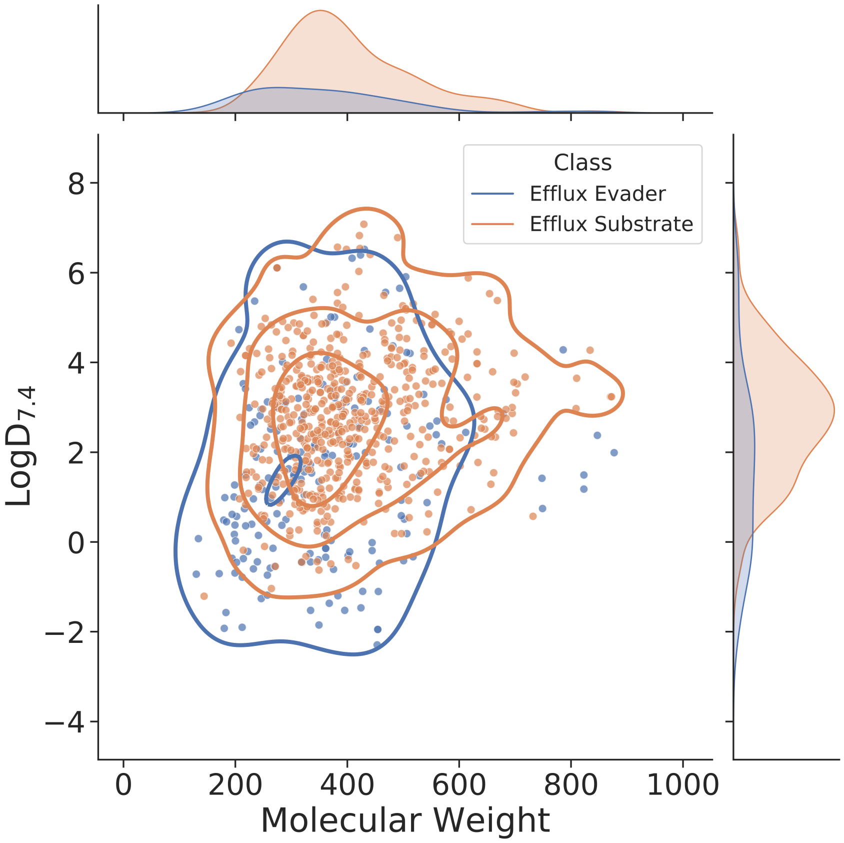

Supplementary Figure 8. Comparison of *LogD* and MW among efflux evaders and substrates show a shift to lower *LogD* and a slight shift to lower MW for efflux evaders.

Cluster	Classes	MCS Evader	MCS Substrate	MCS All
1	Efflux Substrate - 37<br>Efflux Evader - 3	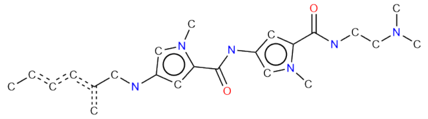	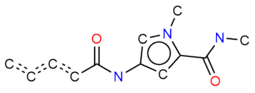	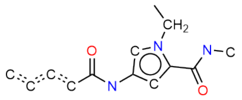
2	Efflux Substrate - 9<br>Efflux Evader - 23	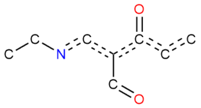	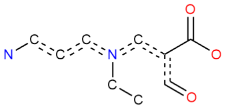	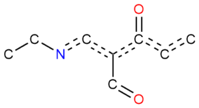

Supplementary Figure 9. Maximum common substructures (MCSs) of two distinct clusters of similar compounds resulting from t-SNE
